# Clear cell renal cell carcinoma combined with intravascular large B-cell lymphoma: a case report and review of the literature

**DOI:** 10.3389/fonc.2026.1788733

**Published:** 2026-02-18

**Authors:** Zhen Zheng, Yuwei Shou, Jingqi Zhou, Enjie Liu, Minglei Yang, Xiu Liu, Jianguo Wei

**Affiliations:** 1 Department of Pathology, Zhejiang Hospital, Hangzhou, China; 2 Department of Pathology, the First Affiliated Hospital of Zhengzhou University, Zhengzhou, China; 3 Department of Pathology, Jiyuan People’s Hospital, Henan, China

**Keywords:** clear cell renal cell carcinoma, clinicopathological features, diagnostic, intravascular large b-cell lymphoma, renal

## Abstract

**Background:**

Clear cell renal cell carcinoma (ccRCC) combined with intravascular large B-cell lymphoma (IVLBCL) is an extremely rare collision tumor, and its coexistence is often missed or misdiagnosed, making clinical diagnosis and treatment notably difficult.

**Case demonstration:**

We present the case of a 68-year-old male, in whom imaging revealed a nodular soft tissue density shadow in the parenchyma of the left kidney. Immunohistochemistry and molecular testing confirmed the combination of ccRCC and IVLBCL. Following surgery, the patient underwent comprehensive examinations, including bone marrow biopsy and cytology, to rule out systemic lymphoma involving the kidneys. The patient received postoperative chemotherapy and was still in a favorable condition after 16 months of follow-up.

**Conclusion:**

In the ccRCC combined with IVLBCL, the lymphoma component is confined within the blood vessels of the RCC, making it extremely prone to misdiagnosis, which can lead to a poor prognosis. This case report presents an incidental finding of ccRCC combined with IVLBCL, aiming to raise physician awareness and provide valuable clinical references.

## Background

Renal cell carcinoma (RCC) has a significant morbidity and mortality rate, accounting for 2% to 3% of all adult malignancies. Clear cell renal cell carcinoma (ccRCC) is the most common histologic subtype, accounting for 85% to 90% of all RCC cases (1). Intravascular large B-cell lymphoma (IVLBCL) is an uncommon and aggressive extranodal large B-cell lymphoma in which malignant B-cells proliferate selectively within the lumina of tiny arteries (2). Intravascular lymphoma (IVL) coexisting with primary benign or malignant tumors is not rare, and has been seen in organs such as the ovary, breast, gastrointestinal tract, and kidney (3). IVLBCL-associated malignancies include ovarian carcinoma (4), breast cancer (5), gastrointestinal stromal tumors (6), meningioma (7), and vascular tumors such as hemangioma, lymphangioma, angiolipoma, and Kaposi sarcoma (4). However, a review of the literature revealed that there are currently no large-scale series reports on IVLBCL combined with ccRCC. Since 2001, only six similar cases have been published in the reports. (**Table 1**, cases 1–6) (3, 8–12).

**Table 1:** Clinical and pathological features of IVLBCL combined with ccRCC including previously (n= 6) and currently (n= 1) reported cases.

Case No.	References	Age/Sex	No. RCC	Size of RCC/ site within kidney	Treatment	Lymphoma in other site	Histology of RCC	Relationship of IVLBCL to RCC	Follow-up (months)
1	Kurek et al. (10)	82/female	1	7.2cm/lower pole right	RRN	Without	ccRCC, ISUP grade 2	Limited to within the RCC	18; Died of cerebral infarction
2	Serrano et al. (3)	72/male	1	4.5cm/right	RRN; A cycle of chemotherapy	Central nervous system	ccRCC, ISUP grade 1	Limited to within the RCC	1; Died
3	Noda et al. (11)	69/female	1	1.7cm/lower pole right	RAPN; 6 cycle of R-CHOP chemotherapy	Involving the normal tissues of the right kidney and the skin, stage IVB IVLBCL	ccRCC, ISUP grade 1	Involving the RCC and the normal tissues of the right kidney	18; Complete remission,ANED
4	Zhang et al. (9)	72/male	1	Not detailed	RRN	Without	ccRCC	Limited to within the RCC	Not detailed
5	Wang et al. (8)	77/female	1	9.5cm/upper pole right	RRN; Combination chemotherapy	Subcutaneous of the breast	ccRCC, ISUP grade 2	The intravascular in the RCC	6; Died of pneumonia
6	Liu et al. (12)	63/male	1	5.0cm/upper Pole left	LRN; Chemotherapy	With bilateral adrenal metastases (clear cell renal cell carcinoma and IVLBCL)	ccRCC, ISUP is not detailed	Limited to within the RCC	13; Died
7	This study	68/male	1	3.0cm/left	RAPN only	Without	ccRCC, ISUP grade 3	Limited to within the RCC	16; ANED

RCC, Renal cell carcinoma; IVLBCL, Intravascular large B-cell lymphoma; ccRCC, Clear cell renal cell carcinoma; RAPN, Robot-assisted partial nephrectory; RRN, Right radical nephrectory; LRN, left radical nephrectory; ANED, indicates alive with no evidence of disease.

In this study, we describe an additional case of IVLBCL associated with ccRCC that was identified incidentally within resected RCC. By reviewing the literature, we explored its clinical, morphological, immunophenotypic, and pathogenic aspects to enhance the comprehensive recognition of the clinicopathological features of this collision tumor and ensure diagnostic accuracy.

## Case demonstration

**Figure 1:**
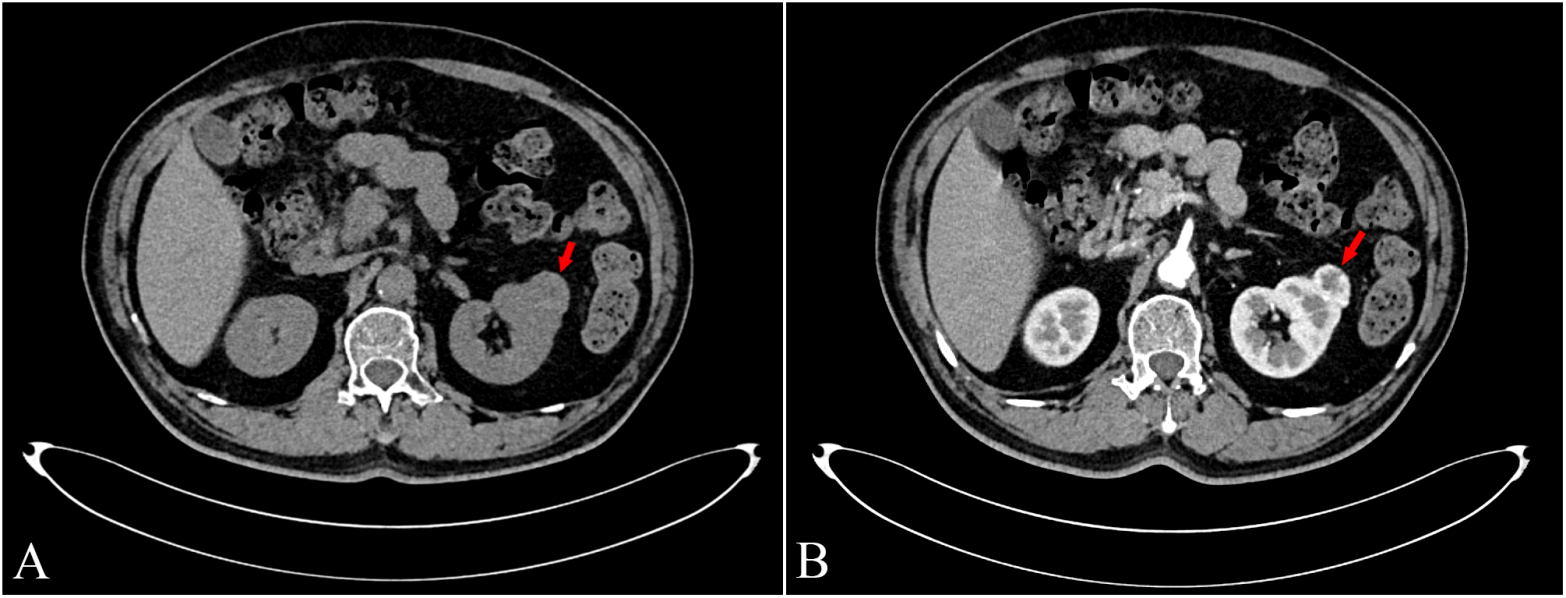
Abdominal contrast-enhanced CT scan of the kidney tumor and tumor macroscopy. An abdominal contrast-enhanced CT scan revealed a soft-tissue density protuberance with a diameter of approximately 28 mm in the left kidney (**A**, red arrow). In the arterial phase, the tumor exhibited slightly uneven enhancement, and the capsule was significantly enhanced (**B**, red arrow).

A 68-year-old man presented to our hospital with cough, dyspnea, and right-sided lower back pain. He explicitly denied nausea, vomiting, hematuria, urinary frequency, urgency, and dysuria. Aside from well-controlled diabetes mellitus, he had no family history of other malignant tumors, and no carcinogenic risk factors were identified. Abdominal contrast-enhanced CT revealed a soft-tissue-density exophytic lesion in the left kidney, measuring approximately 28 mm in diameter (**Figure 1A**). The lesion demonstrated marked enhancement on contrast-enhanced imaging (**Figure 1B**), consistent with RCC. No significant retroperitoneal lymphadenopathy was observed. All hematologic tumor marker assays yielded results within normal reference ranges, with no abnormalities detected. Neither clinical manifestations, nor laboratory findings, nor imaging studies showed evidence of lymphoma.

**Figure 2:**
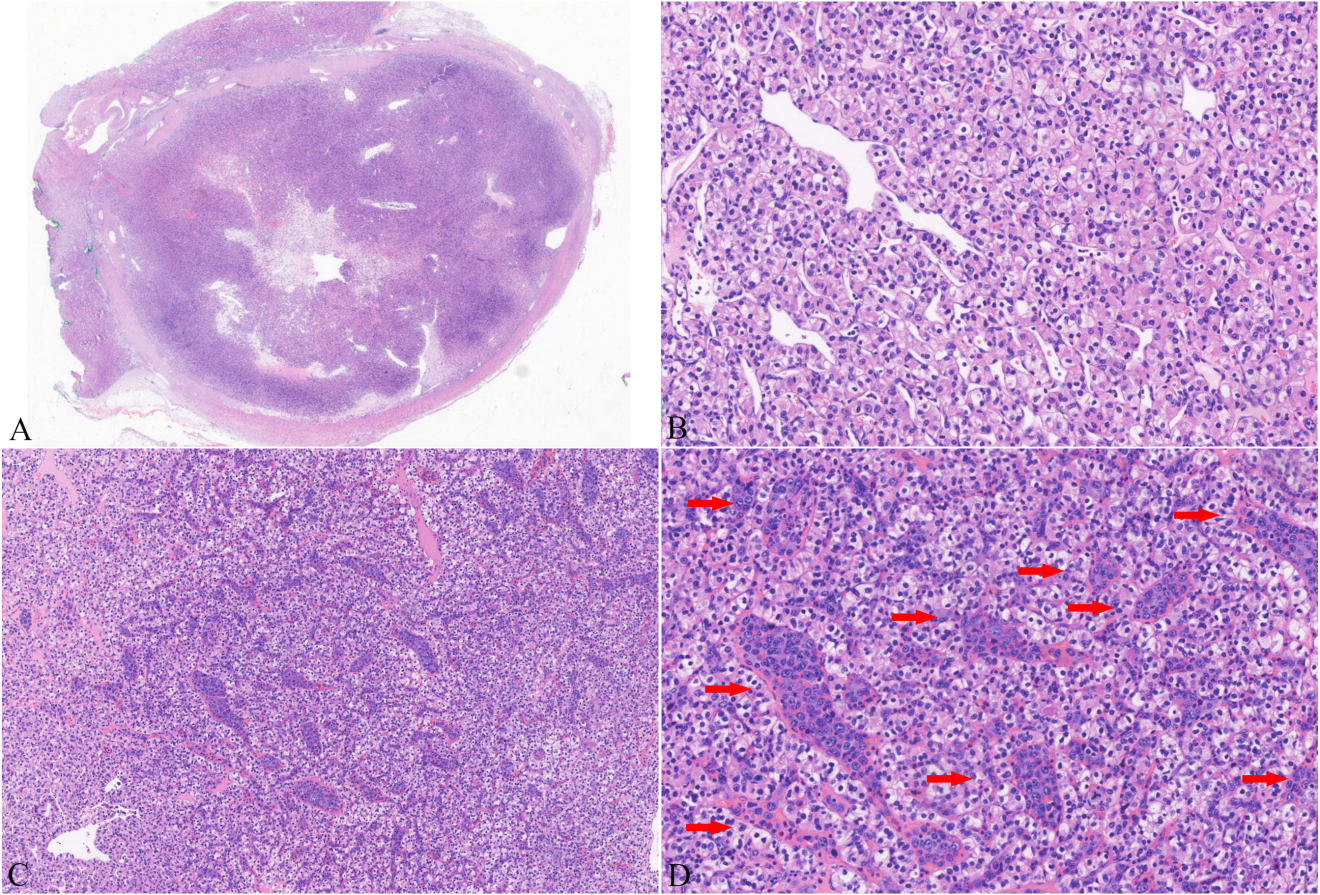
Histology of a representative image from the presented case. Hematoxylin and eosin (H&E) staining showed a tumor encapsulated by a thick fibrous capsule with distinct boundaries **(A)**. Tumor cells exhibited abundant clear to eosinophilic cytoplasm and prominent basophilic nucleoli **(B)**. Large and highly atypical lymphoid cells **(C)** were detected in the small- to medium-sized thin-walled vessels of clear cell renal cell carcinoma (ccRCC). These cells had irregular nuclear outlines, coarse chromatin, prominent single or multiple nucleoli, and scant to moderate cytoplasm **(D)**. (The red arrows indicate the lymphoma component.).

**Figure 3:**
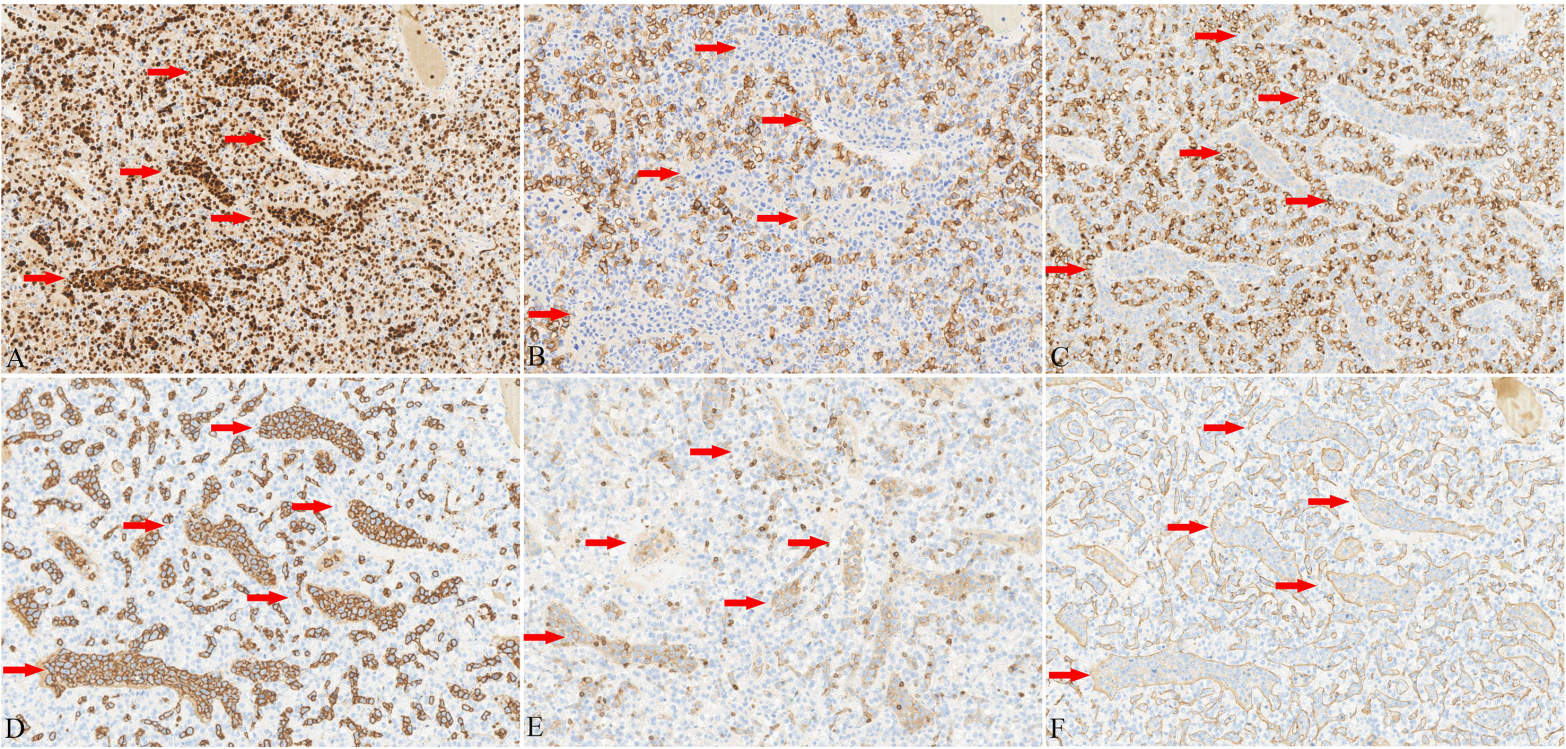
Immunohistochemistry analysis. Immunohistochemistry (EnVision method) showed that ccRCC tumor cells displayed diffuse expression of PAX-8 **(A)**, CA-IX **(B)**, and CD10 **(C)**. In contrast, the atypical lymphoid cells showed diffuse and strong positivity for CD20 **(D)** and PAX-8**(A)**, while CD5 **(E)** showed only weak positivity. Staining of the vascular endothelium with CD34 clearly delineated the intravascular location of the atypical cells **(F)**. (The red arrows indicate the lymphoma component.).

Subsequently, the patient underwent robot-assisted partial nephrectomy (RAPN) of the left kidney. Macroscopically, the RCC was well-circumscribed, presenting a solid, soft, grayish- yellow to grayish-red cut surface. Histological examination showed a tumor surrounded by a thick fibrous capsule with distinct boundaries (**Figure 2A**). The tumor cells had abundant clear to eosinophilic cytoplasm and prominent basophilic nucleoli (**Figure 2B**), and there were no signs of coagulative necrosis, rhabdoid, or sarcomatoid features. A rich capillary network was intertwined with tumor cells, forming the classic “vascular-tumor cell” pattern, which was consistent with conventional ccRCC and showed grade 3 nuclear features according to the World Health Organization (WHO)/International Society of Urologic Pathologists (ISUP) grading system. Moreover, the RCC was limited to the kidney, with no evidence of vascular invasion, perinephric fat involvement, or other aggressive characteristics. Immunohistochemistry demonstrated that the tumor cells expressed AE1/AE3, PAX-8 (**Figure 3A**), CA-IX (**Figure 3B**), and CD10. The Ki-67 proliferation index of the tumor cells was approximately 10%.

Moreover, within the RCC specimens, we identified large, significantly atypical cells within small- to medium-sized, thin-walled vessels. These cells were distributed either individually or in clusters and showed loss of cellular cohesion (**Figure 2C**). The nuclei exhibited irregular contours, coarse chromatin, and one or multiple prominent nucleoli. The cytoplasm was scant to moderately abundant (**Figure 2D**). Consequently, because hematoxylin and eosin staining alone was insufficient to identify the lineage of the atypical cells, we performed immunohistochemical staining for both epithelial and lymphoid lineage markers. The tumor cells showed strong positive immunoreactivity for CD19, CD20 (**Figure 3C**), and CD79a. In contrast, the atypical cells within the vascular lumens were negative for AE1/AE3, CA-IX (**Figure 3B**), and CD10 (**Figure 3D**). They showed diffuse (100%) positivity for PAX8 (**Figure 3A**) and BCL2; focal positivity was observed for MUM1 (70%), Bcl6 (30%), and c-Myc (20%). p53 expression was consistent with wild-type pattern, and CD3 and CD30 were negative. CD5 showed weak but detectable positivity (**Figure 3E**). CD34 staining clearly delineated the intravascular location of the atypical cells (**Figure 3F**). Kappa and lambda light chain analysis revealed no evidence of clonal light chain restriction in the atypical cells. EBER *in situ* hybridization yielded negative results. The Ki-67 proliferation index of the atypical cells was markedly elevated (approximately 80%). Subsequently, fluorescence *in situ* hybridization (FISH) performed on paraffin sections showed no breaks in the BCL6, BCL2, or c-Myc genes. Collectively, these characteristics were consistent with IVLBCL of non-germinal center B-cell (non-GCB) origin. Importantly, the IVLBCL was restricted solely to the ccRCC tumor and did not involve the vessels in the surrounding renal parenchyma.

Post-operative PET-CT demonstrated no abnormal uptake. Examinations of bone marrow aspiration, biopsy, and smear revealed no evidence of lymphoma. Based on the patient’s clinical history, imaging characteristics, histopathological findings, and immunohistochemistry results, the pathological diagnosis was ccRCC (World Health Organization/International Society of Urological Pathology [WHO/ISUP] Grade 3) combined with IVLBCL (Hans classifier, non- germinal center B-cell type). The patient received six cycles of standard rituximab, cyclophosphamide, doxorubicin, vincristine, and prednisone (R-CHOP) chemotherapy after surgery, followed by two cycles of intensified treatment. During and after chemotherapy, the patient underwent intermittent imaging and laboratory examinations, none of which revealed any significant abnormalities. Throughout the 16-month follow-up period after surgery, the patient remained in good health, with no evidence of recurrence or metastasis of ccRCC or IVLBCL.

## Discussion

IVL, a rare extranodal lymphoma, is characterized by the selective proliferation of tumor cells within the lumina of small vessels. B-cell lymphoma constitutes 88% of IVL cases (9). Initially described by Pfleger and Tappeiner (13) in 1958 as “systemic endotheliomatosis,” it was officially defined as IVLBCL in the 2001 WHO classification of tumors of hematopoietic and lymphoid tissues as a distinct subtype of extranodal diffuse large B-cell lymphoma (DLBCL). This definition was maintained in the 2022 5th Edition of the WHO Classification. IVLBCL is extremely rare, with an estimated incidence of 0.095 per million. Lesions can occur at various anatomical locations. The central nervous system and skin are the most frequently affected organs, accounting for 32% and 47% of cases, respectively (8). The kidney is also a target organ for IVLBCL, with reported renal involvement in 21% of cases; nevertheless, IVLBCL restricted only to the kidney is rare (14).

A collision tumor is precisely defined as two histologically distinct malignant tumors that occur simultaneously within the same organ or anatomical space, with no intervening non-neoplastic tissue between the primary tumors (15). This phenomenon has been extensively documented in the scientific literature and can manifest in various anatomical locations, including the gastrointestinal system, lymph nodes, lungs, adrenal glands, thyroid, vertebrae, and meninges (15). Collision tumors in the kidney are infrequent but have been reported, encompassing combinations such as papillary renal cell carcinoma (PRCC) with ccRCC, PRCC with angiomyolipoma, and ccRCC with collecting duct carcinoma (16). The exact incidence rate of IVLBCL within RCC tissue remains undetermined. Based on our institutional archives, among the RCC cases diagnosed during the study period, merely 0.04% were associated with IVLBCL. Moreover, only six relevant reports have been found in the literature (**Table 1**), and the most recent one was published in 2024. All patients underwent surgical intervention for ccRCC and were simultaneously diagnosed with IVLBCL. In Cases 1 and 4, IVLBCL was initially detected solely within the renal cell carcinoma tissue (9, 10). In contrast, after the concurrent diagnosis of IVLBCL and ccRCC in Case 2, IVLBCL involvement was also identified in the central nervous system (3). In Case 3, IVLBCL infiltration was observed not only in the normal renal parenchyma but also in cutaneous tissues (11). Case 5 presented with DLBCL involving the mammary skin concurrently with IVLBCL combined with ccRCC (8). Case 6 demonstrated synchronous ccRCC and IVLBCL in both the kidney and bilateral adrenal glands (12). These findings suggest that, in Cases 2, 3, 5, and 6, IVLBCL may represent metastatic spread or secondary involvement from extrarenal sites—rather than a primary renal origin, although primary renal ccRCC combined with IVLBCL metastasis to bilateral adrenal remains a possible etiology in Case 6. In contrast, Cases 1 and 4 likely represent genuine primary renal IVLBCL, sharing features with our present case.

Including this case, there were a total of 7 cases, comprising three women and four men aged 63 to 82 years (median age: 72 years). Four cases were located in the right kidney, and two were in the left kidney. Both the upper and lower poles of the kidneys could be affected. The treatments consisted of radical nephrectomy (5/7) and robot-assisted radical nephrectomy (2/7, including the present case). All 7 patients had solitary renal cell carcinoma. Among them, 6 cases had a tumor size ranging from 1.7 to 9.5 cm (median size: 5.2 cm). Two cases were cystic and solid, one case had a focal area of hemorrhage, one case presented as polychromatic, and the characteristics of the remaining cases were unknown. Notably, all seven RCCs were ccRCCs. Four of these ccRCCs exhibited low-grade nuclear features according to the WHO/ISUP grading (grade 1 to 2), two cases had unspecified grades, and the present case was ISUP grade 3. The high frequency of ccRCC in this small cohort might not be a coincidence, suggesting that ccRCC might play a role in the coexistence of these two distinct pathological entities, although the small sample size precludes statistical analysis. Follow-up data were available for 6 patients, and the follow-up period ranged from 1 to 18 months (median: 14.5 months). Case 5 (8) underwent chemotherapy (cyclophosphamide, vincristine, doxorubicin, and prednisone) subsequent to surgery but succumbed to pneumonia 6 months postoperatively. Case 2 (3), presenting with concurrent central nervous system (CNS) IVLBCL, passed away within one month after one cycle of hyper-CVAD chemotherapy. Case 1 (10) did not receive chemotherapy following surgery. One year later, she was diagnosed with large cell lung cancer and died of cerebral infarction 18 months after surgery. Case 6 (12) received chemotherapy after surgery and died 13 months after follow-up. Case 3 (11), diagnosed with concurrent stage IVB IVLBCL, received six cycles of R-CHOP chemotherapy, achieved complete remission, and showed no recurrence of ccRCC or IVLBCL 1.5 years after surgery. Similar to but distinct from this situation, in our case, even though no signs of IVLBCL were detected in other parts of the body, the patient underwent R-CHOP chemotherapy after surgery and remained in good health 16 months postoperatively.

As is widely known, IVLBCL is an aggressive variant of DLBCL with a dismal prognosis. The most recent revised classification of hematopoietic and lymphoid tissue tumors by the WHO proposes that IVLBCL should be categorized into three subtypes according to clinical features: the classic subtype, the cutaneous subtype, and the subtype associated with hemophagocytic syndrome (17). Classic IVLBCL typically manifests with nonspecific symptoms such as fever, fatigue, and alterations in consciousness. In the cutaneous subtype, lesions are restricted to the skin, and the disease progresses at a slower pace. The subtype related to hemophagocytic syndrome has the most rapid onset and progression. In this particular case, since the patient’s condition was limited to the kidneys, the classic subtype was considered the most appropriate. Nevertheless, the preferred treatment option for all subtypes of IVLBCL is the cyclophosphamide, doxorubicin, vincristine, and prednisone (CHOP) regimen. It has been documented that the overall response rate for IVLBCL patients treated with the CHOP regimen was 59%, and 33% of the patients achieved a 3-year overall survival rate. Incorporating rituximab into the CHOP regimen (R-CHOP) led to a complete response rate of 88%, an overall response rate of 91%, and a 3-year overall survival rate of 81% among the patients (17). Furthermore, it has been found that CD5-positive IVLBCL is more prone to affect the liver, spleen, and bone marrow, potentially triggering hemophagocytic syndrome, and is associated with a worse prognosis (13). In this case of IVLBCL, the tumor cells display weak expression of CD5. Although there has been no recurrence in the short term, it remains unclear whether this will lead to a less favorable prognosis in the future. Further observation is necessary.

The mechanism underlying the concurrent occurrence of IVLBCL and ccRCC remains poorly understood. Rafael et al. (18) summarized that the prevailing hypotheses regarding collision tumors are as follows: (i) Malignant lymphocytes might cause cytokine imbalance or undermine immune surveillance, thereby diminishing the capacity to induce apoptosis and suppress tumor cell proliferation, potentially leading to the development of solid tumors; (ii) solid tumors, which are highly vascularized and can secrete growth factors, may provide a favorable microenvironment for the proliferation of malignant lymphocytes; (iii) lymphomas could emerge as a result of a chronic immune response to solid tumors; and (iv) both tumor types may share common etiological factors, such as Epstein-Barr virus (EBV), Helicobacter pylori, or murine mammary tumor virus. Additionally, some researchers have proposed that these tumors may arise through distinct molecular pathways and coexist coincidentally (15). It has been reported that the potential interactions between solid tumors and lymphomas, involving inflammatory and immunosuppressive mechanisms, may contribute to tumor progression and dissemination (18). Moreover, the specific mechanism requires further large-scale data analysis and molecular research.

Histologically, it is of utmost importance to differentiate IVLBCL in ccRCC from high-grade transformation or dedifferentiation of RCC. As indicated in this series of collision tumors, the RCC component generally does not display a high nuclear grade. Conversely, the atypical cells in IVLBCL are large, hyperchromatic, and significantly pleomorphic, which might potentially be mistaken for high-grade RCC. Therefore, a meticulous assessment of morphological features with the assistance of immunohistochemical markers is necessary to distinguish neoplastic large B cells from RCC cells. IVLBCL typically expresses B-cell markers such as CD19, CD20, and CD79a. Some GCB type IVLBCL may express CD10 but usually do not express epithelial markers or CA-IX. It is noteworthy that certain types of lymphoma can express the PAX8 protein (19, 20). As demonstrated in this case, lymphoma cells within blood vessels can also express PAX8, rendering them susceptible to being misdiagnosed as poorly differentiated renal cell carcinoma. Secondly, ccRCC combined with IVLBCL frequently demonstrates a biphasic cellular morphology. Nevertheless, other RCC subtypes, including chromophobe RCC, TFEB-rearranged RCC, and biphasic squamoid alveolar RCC, can also display biphasic patterns and might mimic ccRCC combined with IVLBCL. However, these tumors possess distinct morphological characteristics. For example, TFEB-rearranged RCC is characterized by a typical biphasic pattern, in which larger clear cells encircle nests of smaller cells that are often accompanied by basement membrane-like material and calcifications (21). Moreover, these biphasic RCCs generally do not have the accumulation of neoplastic B-cells within the lumina of small- to medium-sized vessels, a feature observed in IVLBCL. Additionally, immunohistochemistry (IHC) can effectively differentiate IVLBCL (positive for CD19, CD20, and CD79a) from biphasic RCCs (positive for cytokeratin and PAX8). In addition, when handling morphologically abnormal cell populations in low-grade RCC, we need to take into account the possibility of “tumor-to-tumor metastasis.” RCC has been reported as one of the most prevalent “recipient” tumors for metastases, while lung and breast cancers are frequent “donor” tumors (22). However, metastatic carcinoma is typically positive for cytokeratin and negative for LCA, and a known primary tumor is usually present in the patient. While IVLBCL is positive for LCA, expresses B-cell markers, and has no known primary carcinoma. Finally, it is essential to carefully differentiate it from DLBCL involving the vessels and lymphatics. Some evidence indicates that IVLBCL may be an aggressive variant or an intravascular pattern of recurrence/progression of DLBCL, with a poor prognosis (13, 22), although the exact mechanism remains unclear. Moreover, primary CD5-positive DLBCL often exhibits partial intravascular or intrasinusoidal infiltration, which histologically mimics IVLBCL (23).

## Conclusion

In summary, we present a case of ccRCC combined with IVLBCL. Failure to detect IVLBCL in the resected RCC tissue might result in a misdiagnosis of high-grade transformation or biphasic RCC. Moreover, IVLBCL is an aggressive lymphoma, and its poor prognosis is frequently exacerbated by delayed diagnosis. Therefore, pathologists should heighten their awareness of this tumor type to avoid missed diagnoses and guarantee timely treatment for patients. The exact pathological mechanisms behind the co-occurrence of ccRCC and IVLBCL remain elusive. Further case observations and molecular studies are necessary to clarify whether the two tumors develop independently, share common causative factors, or interact with each other.

## Data Availability

The original contributions presented in the study are included in the article/supplementary material. Further inquiries can be directed to the corresponding author.
